# Fate and Transport of *Toxoplasma gondii* Oocysts in Seasonally Snow Covered Watersheds: A Conceptual Framework from a Melting Snowpack to the Canadian Arctic Coasts

**DOI:** 10.3390/ijerph10030994

**Published:** 2013-03-11

**Authors:** Audrey Simon, Michel Bigras Poulin, Alain N. Rousseau, Nicholas H. Ogden

**Affiliations:** 1 Groupe de Recherche en Épidémiologie des Zoonoses et Santé Publique, Faculté de Médecine Vétérinaire, Université de Montréal, 3200 Sicotte, CP 5000, Saint-Hyacinthe, J2S 7C6, Québec, Canada; E-Mails: michel.bigras.poulin@umontreal.ca (M.B.P.); nicholas.ogden@phac-aspc.gc.ca (N.H.O.); 2 Institut National de Recherche Scientifique, Centre Eau Terre Environnement, 490, rue de la Couronne, Québec, G1K 9A9, Québec, Canada; E-Mail: alain.rousseau@ete.inrs.ca; 3 Zoonoses Division, Centre for Foodborne, Environmental and Zoonotic Infectious Diseases, Public Health Agency of Canada, 3200 rue Sicotte, Saint-Hyacinthe, J2S 7C6, Québec, Canada

**Keywords:** estuary, faeces-borne pathogen, hydrological transport, large-scale dispersion, snowmelt runoff, *Toxoplasma gondii*, waterborne contamination

## Abstract

*Toxoplasma gondii* is a zoonotic protozoan that causes serious illness in humans and infects animals worldwide, including the Canadian Arctic. Indeed, high prevalence of infection amongst Inuit has been recorded, possibly due to consumption of raw infected seal meat. Here we explore the hypothesis that *T. gondii* oocysts contaminate the coastal marine environment via surface runoff from across the boreal watershed, particularly during the snowmelt period. We propose a conceptual framework of the different processes governing the fate and transport of *T. gondii* oocysts from the melting snowpack to the Canadian arctic coast via the freshwater runoff. This framework identifies the feasibility of a transmission pathway of oocysts from contaminated soil to the marine environment, but also the complexity and multiplicity of mechanisms involved. In addition, the framework identifies knowledge gaps for guiding future studies on *T. gondii* oocysts. Furthermore, this work could be used as a tool to investigate the possible estuarine contamination by other faeces-borne pathogens transported via the spring freshet in seasonally snow covered watersheds.

## 1. Introduction

To prevent coastal contamination of pathogens via freshwater runoff, an understanding of the transport and fate of microbes at the watershed scale is needed. Numerous experimental studies have investigated the survival of pathogens under various environmental conditions in the laboratory, whereas relatively little is known about their survival and transport in the real environment [1]. Ferguson [1] reviewed the processes involved in the transport of pathogens in surface water and proposed a conceptual framework to study and to fill the gaps in our knowledge. Water flow is one of the most important parameters affecting transport of pathogenic microbial contaminants from the terrestrial environment. Outbreaks of disease due to these pathogens occur particularly after large hydrological events such as intense rainfall that dislodges them from their terrestrial locations, and results in their transport by runoff water to contaminate food or drinking water supplies [1,2,3]. *Toxoplasma gondii*, the protozoal agent of toxoplasmosis is no exception; outbreaks of disease have been associated with *T. gondii* oocysts released from the environment into drinking water supplies following heavy rainfall events [4]. The relationship between the release of microorganisms and snowmelt is, however, less recognized, but it too can remobilize microorganisms stored in the soil from the previous summer and carry them into streamflow [5,6]. In addition, snowpack could accumulate and concentrate some pathogens shed during winter, which would also be discharged into streams when the short melt period occurs. The mechanisms underlying release and dispersion of accumulated microorganisms in snowmelt have receive little attention in the literature, while the release of organic contaminants from the snowpack is recognized to represent significant risks to aquatic and terrestrial organisms in seasonally snow-covered watersheds [7].

In the Canadian Arctic, there is recent serological evidence for *T. gondii* infections in marine mammals [8], which are also exposed to this parasite in other parts of the World [9]. These marine mammals may serve as a source of *T. gondii* infection for Inuit people that consume raw infected seal meat, making toxoplasmosis a significant public health threat for Inuit in the Canadian Arctic [8]. *T. gondii* can cause serious illness in humans, particularly in immunocompromised people and congenitally infected children, resulting in neurologic disorders and foetal death and abortion [9]. Domestic cats and wild felids are the only known definitive hosts capable of shedding *T. gondii* oocysts in their faeces [9]. The occurrence of infection in marine mammals is, therefore, intriguing but it suggests that the most likely source of their infection is contamination of the marine environment from a terrestrial source. Recent evidence from California suggests that the parasite can cross from terrestrial to marine ecosystems via freshwater runoff, and this pathway of transmission could explain the high incidence of toxoplasmosis in southern sea otters (*Enhydra lutris* *nereis*) [10,11,12]. Furthermore, the detection of *T. gondii* in one mussel collected along the California coast just after a significant rainfall event suggests the significance of precipitation-excess runoff in the exposure of coastal organisms to *T. gondii* [13]. Spring conditions in the seasonally snow-covered watersheds such as the Canadian Arctic watershed are marked by a very large volume of freshwater runoff due to snowmelt and rainfall. The rapid release of oocysts into rivers at this time, after they have accumulated for months in the soil and snowpack, could represent a significant contamination process of the arctic coastline.

The mechanisms of transport and the fate of *T. gondii* oocysts in the environment are largely unknown. The objective of this review is to develop a conceptual framework for investigating the different processes behind the fate and transport of *T. gondii* oocysts to the Canadian arctic coast via the snowmelt runoff directly or indirectly (via infected food animals) causing disease in humans. More specifically, the focus is in the potential factors that could influence the occurrence of high concentrations of pathogens in the Canadian Arctic estuarine environment, which would be expected to increase the probability of infection of aquatic organisms and food obtained from the coastal Arctic environment.

## 2. The Framework

As reviewed by VanWormer *et al.* [14], several factors influence the *T. gondii* oocysts-based in terrestrial and aquatic environments.

**Figure 1:**
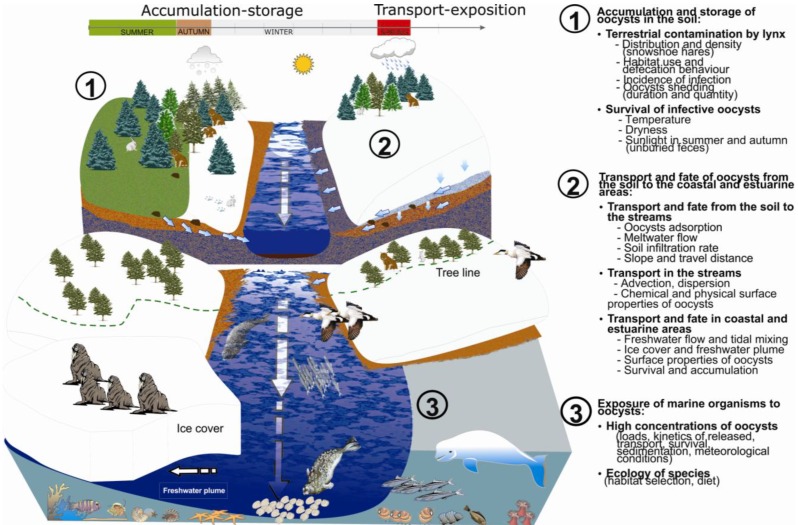
Watershed cross-section diagram showing the fate and transport of *Toxoplasma gondii* oocysts by the snowmelt runoff from the headwater to the estuary within the Canadian Arctic watershed.

Specifically, Figure 1 illustrates the different processes that could lead to estuarine contamination by *T. gondii* oocysts in the Canadian Arctic watershed. These processes, occurring on several spatio-temporal scales, are discussed in the following sections from the perspective of *T. gondii* oocysts but are likely applicable in many cases to faecally-excreted oocysts of other pathogenic protozoa.

### 2.1. Accumulation and Storage of T. gondii Oocysts in the Soil and within the Snowpack

#### 2.1.1. Environmental Contamination by *T. gondii* oocysts

After primary infection, a single domestic cat or wild felid may shed millions of *T. gondii* oocysts in faeces for about one week of its life [9]. *T. gondii* oocysts are subspherical to spherical in shape and 10 × 12 μm in size and have a highly resistant and impermeable wall [9]. In boreal forest covering a great part of the Canadian Arctic watershed, a recent study by Simon *et al.* [15] revealed that populations of Canadian lynx (*Lynx canadensis*) may be the main source of environmental contamination with *T. gondii* oocysts (Figure 1). Following primary infection (most likely from their main prey, the snowshoe hare *Lepus americanus*), lynx would shed, like other newly-infected felids, large amounts of unsporulated oocysts in their faeces, which need to sporulate to become infectious and environmentally resistant [9]. While there are no data on *T. gondii* infection in snowshoe hare, other species of hare are commonly infected [9,16]. The level of environmental contamination by oocysts would vary according to the density of lynx and the incidence of infection in lynx populations [15]. Spatial variations of *T. gondii* contamination by lynx would be expected to depend on their distribution at the population level, and on habitat use and behaviour of defecation at the individual level. 

#### 2.1.2. Survival of *T. gondii* Oocysts

A key feature to the biology of *T. gondii* is the resistance and persistence of oocysts. Very low sub-zero temperatures (<−20 °C), sunlight (particularly ultraviolet light [UV]) and dryness are environmental stressors that have been identified as key factors for inactivation of protozoan pathogens in general [1] and can be lethal for *T. gondii* oocysts in particular [17,18]. The soil and snow environments of excreted oocysts may provide refuge from extremes of temperature, sunlight and desiccation [19]. Particularly, snowpack acts as an insulator, protecting the ground surface from the far sub-zero air temperatures experienced in the Canadian Arctic [20]. However, very little is known on the unsporulated oocysts’ ability to survive and sporulate in diverse natural conditions. Experimentally, some oocysts were able to sporulate and to remain infective for mice after storage for three months at 4 °C [21] and unsporulated oocysts were killed by 1 to 7 days of constant freezing (at −6 °C) [17]. This latter study suggests that freezing conditions within snowpack may prevent winter-shed oocysts in northern latitudes from sporulating (and so becoming infectious and environmentally resistant). Therefore, accumulation of *T. gondii* oocysts may be most important in spring, summer and early autumn, when temperatures are suitable for sporulation. Furthermore, spring-born lynx kittens may be the most significant age group causing environmental contamination by *T. gondii* oocysts, as the onset of their hunting activity and territory exploration behaviour expose them to their first exposure to *T. gondii* [22]. After sporulation, *T. gondii* oocysts could accumulate in soil and survive through the winter under the snowpack. Indeed, all studies underline the remarkable resistance of sporulated *T. gondii* oocysts to direct exposure to extremely cold temperatures. Experimentally, sporulated *T. gondii* oocysts are able to remain infectious after 28 days at −21 °C [17] and 106 days at temperatures ranging from −5 °C to −10 °C [23], which suggests that oocyst infectivity is not eliminated by freezing. Under natural conditions, oocysts were able to keep their infectivity for 18 months through two cold winters in Kansas (minimum temperatures below −20 °C) [24]. To our knowledge, only one study has quantified the temporal viability of *T. gondii* oocysts at stable room temperatures, suggesting that a decreasing of oocyst load of 2 to 6 log units could be expected over winter [25]. This type of information may be more reliable for quantifying the number of infective oocysts able to contaminate surface water. Additional to environmental effects on the survival of oocysts, lynx defecation behaviour may influence the number of oocysts that can reach surface water. Indeed, lynx mark their territory by leaving their faeces uncovered [22], exposing them of deleterious effect of air temperature, dryness and sunlight. However, even if oocysts in buried faeces were protected, their spreading and then their delivery to streams, could be altered by interactions with soil particles [14]. Globally, further studies on the viability of oocysts under the environmental conditions in snow covered watersheds, including effects of sunlight (in summer and autumn) and dryness as well as temperature, are needed. In the design of such studies, it may be worth considering the strains of *T. gondii* used; experimental studies tend to use strains of *T. gondii* from temperate zones [17,21,23,24], and given the wide latitudinal footprint of *T. gondii* we speculate that strains adapted to cold (or very hot) environmental conditions may exist.

### 2.2. Transport and Fate of T. gondii Oocysts

#### 2.2.1. Overland Transport from the Soil to the Streams

Transport processes that control pathogen movement in watersheds are determined by adsorption/desorption processes, hydrological, mechanical, and biological movements [1]. Thus, during snowmelt, the attachment of *T. gondii* oocysts to faecal matter and soil particles, as well as the water flow and soil characteristics, likely govern the transport and survival of oocysts from the refugia where they have accumulated to the river network. Particularly, soil infiltration rates determine whether meltwater flows laterally as surface flow, subsurface flow or whether it percolates deeper into the soil [7]. In the case of water-saturated soils, the oocysts could be directly transported into streams via surface flows [6]. Shapiro *et al.* [26] found that oocysts were negatively charged and hydrophilic in freshwater, which may limit their aggregation to other particles and could enhance their transport in the meltwater to river network. Conversely, if the soil is unsaturated (in early spring), it may absorb the meltwater and then it could provide a potential, natural, filtering action and adsorption site for the removal of oocysts, decreasing the probability that they reach streams. In this case, the snowmelt could behave like a light rainfall which tends to reduce terrestrial contamination with micro-organisms without causing contamination of streams [6]. Whether or not *T. gondii* oocysts are adsorbed onto particles or removed by straining through the soil is unknown. Indeed, the extent of oocyst adsorption to soils of related pathogenic protozoa such as *Cryptosporidium* and *Giardia* spp. is still a subject of controversy in the literature [27]. Mass flow and direction may determine the soil sorption and desorption of oocysts [28]. Therefore, during a high flow period such as the snowmelt period, oocysts could move through the water-saturated soil without restriction to nearby surface water [29]. 

The spatial pattern of faecal deposition, associated with the defecation behaviour of lynx, would determine the potential for oocysts to be transported to streams. In particular, the proximity of contaminated faeces to a river network would determine the distance oocysts must travel to reach surface waters and, thus, the likelihood that they get there. Topography of contaminated land may also have significant effects on the transport of oocysts, with increasing elevation and slope being associated with an increase in their capacity for overland transport. Slope of soils and distance over which microorganisms are delivered to the stream have been identified as key transport parameters for faecal coliforms and *Cryptosporidium* [6,28,30,31]. 

#### 2.2.2. From the Streams to the Coastal and Estuarine Areas

The transport of oocysts in the aquatic environment is a result of advection, dispersion, and inactivation (by mortality or settling), and those processes may be strongly driven by the physical properties of oocysts, and particularly by their surface properties [32], which drive their interactions with the water and particles. When released into the river network, the *T. gondii* oocysts may be readily and rapidly transported by the high spring flow because of their buoyancy in fresh water due to their hydrophilic nature and negative surface charge [26]. Therefore, their flocculation, adsorption, filtering out by vegetation and sedimentation are probably negligible in the rapid spring water flow. Furthermore, while Shapiro *et al.* [33] identified water vegetation as a means of oocyst removal from the water through straining and adhesion processes, the low levels of vegetation in watercourses in northern latitudes in early spring may allow oocysts to move in the water column of rivers with limited load loss. 

At the estuary-seawater interface, the vertical distributions of oocysts in the water column may be influenced by the interaction of high freshwater flow at the river mouth, tidal mixing with saline water, and wind forces which determine the salinity gradient in the estuary [34]. As salinity increases, the oocyst surface charge becomes neutral, which leads to efficient aggregation of oocysts with other particles, and ensuing sedimentation in the more saline environment [26,35]. During periods of high stream flow, turbulent mixing in the estuarine areas, which could inhibit sedimentation, results in freshwater plumes that are observed extending from river mouths to spreading along the coasts [34]. The presence of ice cover in the Arctic coastal environment during the spring freshet may enhance the spreading of oocysts. Indeed, ice cover prevents wind and wave actions from causing mixing of salt and fresh waters, which limits the salinity in the plumes [34], and may delay the sedimentation of oocysts. 

Experimentally, sporulated *T. gondii* oocysts can remain infectious for mice for at least two years [36,37]. The survival of oocysts in sediments is unknown, but could be increased as the adsorption of pathogens to particles in the sediment may reduce deleterious effects of UV radiation [1]. A greater survival of *T. gondii* oocysts in this medium could allow them to accumulate over long periods and potentially to be re-suspended in the estuaries as changes in river discharge rate occur, as has been hypothesized for oocysts of other protozoa [1].

### 2.3. Exposure of Marine Organisms to *T. gondii* Oocysts

#### 2.3.1. Meteorological Conditions and Concentrations of *T. gondii* Oocysts in Estuaries

Rates of exposure and infection of estuarine fauna would be expected to be higher when concentrations of protozoal oocysts in runoff are high, and that concentration would likely be influenced by all factors discussed in the previous sections: (i) *T. gondii* oocyst accumulation and load within the watershed; (ii) the kinetics of oocyst release from soils to streams; (iii) transport and survival within streams; and (iv) spreading and sedimentation within the estuarine/coastal environment. Meteorological conditions will likely influence all these processes by affecting the volume of runoff via the amount of total precipitation (accumulated snow and spring rainfall), and the intensity and duration of snowmelt via effects of temperature. Fast melting of snow at the watershed scale that leads to more homogenous release of oocysts via efficient leaching from soil may increase the burden of oocysts that enter streams and deposits in the estuarine environment [7]. Wind and wave-induced turbulence of water may influence the distribution of oocysts within the water column, as well as fresh water-sea water mixing in the coastal areas, which may impact oocyst distribution within the coastal ecosystem and accordingly affect the availability of oocysts for contamination of the coastal organisms.

#### 2.3.2. Ecology of Marine Species and Dietary Pathway

In spring, strong freshwater runoff in some estuaries draining the Canadian Arctic watershed stimulates upwelling of nutrient-rich water, leading to a high biological productivity, which attracts a high diversity and abundance of organisms in those areas [34]. If the distribution and the concentration of oocysts in the water column in the estuarine and coastal environments vary according to the aforementioned physical and biological processes, the organisms living in those areas may be differentially exposed to oocysts depending on their habitat selection within the water column. Marine mammals could become directly infected via drinking water or more likely via the consumption of their prey, in which accumulation of *T. gondii* oocysts could occur. Sedimentation of oocysts carried into the Arctic coastal marine environment would mean that they likely accumulate at the seabed where molluscs thrive. Ectothermic invertebrates exposed to coastal freshwater runoff are strongly suspected to be the source of *T. gondii* infection of Californian southern sea otters (*Enhydra lutris nereis*), and the consumption of filter feeding molluscs and marine snails has been associated with increased risk of *T. gondii* exposure [12,13,38]. Recently, a case-control study identified eating raw oysters, clams, or mussels as a new risk factor for recent *T. gondii* infection in the United States [39], suggesting that consumption of shellfish is a substantial source of infection. In the Canadian Arctic, several species of pinnipeds, cetaceans or birds can feed on the benthic fauna including bivalves, which have been shown to remove *T. gondii* oocysts from contaminated water through filter-feeding activity [40,41]. Furthermore, oocysts can survive and remain infectious to mice for up to three days in mussels (*Mytilus galloprovincialis*) and three months in oysters (*Crassostrea virginica*) [40,42]. In the field, *T. gondii* has to date only been detected in a wild California mussel (*Mytilus californianus*) [13], but the related protozoans *Giardia* and *Cryptosporidium* have been found in marine waters and shellfish worldwide as reviewed by Fayer *et al.* [43]. Like bivalves, fish could plausibly filter *T. gondii* oocysts out of column water making them an efficient vector for piscivorous marine mammals [44]. Moreover, some fish species living in both freshwater, where they are particularly exposed to oocysts, and saltwater environments, could then act as paratenic hosts between the rivers and estuarine or sea areas. Considering that very low (as low as one oocysts [45,46]) parasite doses are needed to infect animals, whose flesh may then remain infective to humans life-long, it is highly plausible that very low concentrations of contaminating oocysts could have significant public health risk. Seals may amplify that risk, one oocyst becoming thousands of tissue cysts.

## 3. Conclusions

Here we have identified multiple processes, occurring at different spatio-temporal scales, to build a conceptual framework for the underlying infection of marine mammals in the Canadian Arctic by *T. gondii*. This framework underlines the potential complexity of processes involved in the fate and transport of *T. gondii* oocysts (and potentially of other terrestrial pathogens) in the snowmelt runoff. Multiple physical and biological processes interact at the micro, macro or watershed scales, mainly depending on the hydrological spring event and the ecology of the parasite and its hosts. More specifically, this framework raises the possibility that snowmelt runoff contaminated by oocysts accumulated in the soil could be a potentially significant source of exposure of aquatic organisms living along the Arctic coastlines in the spring time. Key to this framework is that the resistance and long-term survival of oocysts, their capacity for transport in watersheds via overland and stream flows, and the short and intense event of the high snowmelt flow in the Canadian Arctic, could well enable this transmission route. Furthermore, the seasonal cycle of ecosystems, populations and individual organisms may be important in the magnitude and timing of exposure to *T. gondii* oocysts.

Globally, the fate and the transport of *T. gondii* oocysts at the watershed scale are completely unknown, mainly because there is a lack of information on their transport properties, survival, and prevalence in the environment. Methods to recover and detect oocysts in the aquatic environment exist, but currently, the lack of rapid, practical and sensitive methods still makes their detection challenging [47]. In addition to limitations of our knowledge on the fate and transport of pathogens within watersheds [1], this paper identifies critical points, which are needed to be investigated to further understand the transmission pathways of protozoal oocysts (and perhaps other faecally-transmitted pathogens) by snowmelt runoff. As a first step, there is a need to assess the magnitude of terrestrial contamination by estimating the oocysts shedding prevalence and intensity by the lynx [48]. The isolation of *T. gondii* oocysts from lynx faeces could allow assessment of their capacity to sporulate, survive and remain infective in a cold environment and determine to what extent the load of infectious oocysts decrease over winter. Moreover, the genetic characterisation of *T. gondii* strains would be valuable to track oocysts from shedding in lynx living in the boreal ecosystems to aquatic organisms living in the Canadian Arctic coasts. Also, more data are needed on how aquatic organisms are exposed to *T. gondii* oocysts, especially on the potential mechanisms linking foraging behaviour with the probability of *T. gondii* infection.

Finally, this work raises the question of estuarine contamination via processes occurring over large spatio-temporal scales by faeces-borne pathogens transported by the spring freshet in the seasonally snow-covered watersheds. For persistent pathogens that have wide host species ranges (*i.e*., are host generalists) and/or are shed in the environment in large quantities, the contamination of streams via snowmelt runoff could be very significant. Diffuse or non-point source contamination of the environment by wildlife populations could be a relatively significant contributor to faecal contamination in pristine watersheds that are not impacted by human activities. Largely understudied and often neglected, the faecal contamination by wildlife is difficult to define and quantify, and, hence, represents a significant challenge for further research on contamination of watersheds [48].
